# Exploring Physicians’ Views, Perceptions and Experiences about Broad-Spectrum Antimicrobial Prescribing in a Tertiary Care Hospital Riyadh, Saudi Arabia: A Qualitative Approach

**DOI:** 10.3390/antibiotics10040366

**Published:** 2021-03-31

**Authors:** Nada A. Alsaleh, Hussain A. Al-Omar, Ahmed Y. Mayet, Alexander B. Mullen

**Affiliations:** 1 Department of Pharmacy Practice, College of Pharmacy, Princess Nourah bint Abdulrahman University, Riyadh 84428, Saudi Arabia; naaalsaleh@pnu.edu.sa; 2 Department of Clinical Pharmacy, College of Pharmacy, King Saud University, Riyadh 11451, Saudi Arabia; halomar@ksu.edu.sa (H.A.A.-O.); iymayet@ksu.edu.sa (A.Y.M.); 3 King Khalid University Hospital, Riyadh 11451, Saudi Arabia; 4 Strathclyde Institute of Pharmacy and Biomedical Sciences, University of Strathclyde, 161 Cathedral Street, Glasgow G4 0RE, UK

**Keywords:** qualitative research, broad-spectrum antimicrobial, physicians, prescribing behaviour

## Abstract

Antimicrobial resistance (AMR) is a global public health threat associated with increased mortality, morbidity and costs. Inappropriate antimicrobial prescribing, particularly of broad-spectrums antimicrobials (BSAs), is considered a major factor behind growing AMR. The aim of this study was to explore physician perception and views about BSAs and factors that impact upon their BSAs prescribing decisions. Qualitative semistructured telephone interviews over an eleven-week period were conducted with physicians in a single tertiary care hospital in Riyadh, Saudi Arabia. Purposeful and snowball sampling techniques were adopted as sampling strategy. All interviews were audio recorded, transcribed verbatim, uploaded to NVivo^®^ software and analysed following thematic analysis approach. Four major themes emerged: views on BSAs, factors influencing BSA prescribing and antimicrobial stewardship: practices and barriers and recommendations to improve appropriate BSA prescribing. Recommendations for the future include improving clinical knowledge, feedback on prescribing, multidisciplinary team decision-making and local guideline implementation. Identification of views and determinants of BSA prescribing can guide the design of a multifaceted intervention to support physicians and policymakers to improve antimicrobial prescribing practices.

## 1. Introduction

Antimicrobial resistance (AMR) is a global public health threat associated with increased mortality, morbidity and costs [1]. Inappropriate antimicrobial prescribing, particularly of broad-spectrums antimicrobials (BSAs), is considered a major factor behind growing AMR [2]. Implementing antimicrobial stewardships programmes (AMS) aimed to enhance the appropriate prescribing of antimicrobials may lower inappropriate or overuse of BSAs [3,4]. AMS have shown to decrease the inappropriate antimicrobial use, slow the development of AMR, reduce the length of hospitalisation and the health care-associated costs treating infectious disease [5]. The World Health Organization has recognised the potential of these programmes through endorsement of the antimicrobial stewardship policy and strategy as part of the global action plan to decrease AMR risk. [6] Consequently, implementation of AMS in a hospital setting has been endorsed by several countries and institutions to improve the appropriate use of antimicrobials, thus decreasing AMR [7,8]. Physicians are supportive of AMS [9]; nevertheless, one of the challenges and barriers to AMS is the lack of physician consensus on what is considered an appropriate choice when deciding to initiate prescribing of a BSA [10]. Furthermore, physician perception and views about antimicrobial use and AMR vary across different settings and countries [11,12]. A systematic review of studies on antimicrobial use in hospital settings reported that AMS sustainability and durability can be enhanced with a better consideration and understanding of determinants and elements of antimicrobial prescribing [13]. Cultural, behavioural and contextual factors must be recognised and identified to positively impact on antimicrobial use [14]. To implement successful interventions to improve the appropriateness of BSA prescribing, it is essential to investigate and recognise physicians’ perceptions and views about BSAs and identify how they decided on antimicrobial prescribing [15]. Several qualitative studies on perceptions and views about antimicrobials prescribing have been reported [15,16]. However, little is known about why and how physicians make decisions to prescribe classes of antimicrobial agents, specifically BSAs. Moreover, there are no known studies conducted in Saudi Arabia exploring how differences in health care structures and policies may impact upon physicians and their antimicrobial prescribing habits. Therefore, this study aims to explore physician perceptions and views about BSAs and factors that impact upon their prescribing decisions.

## 2. Results and Discussion

Sixteen physicians agreed to participate in the study. Their mean age was 30.6 ± 5.80 years old, and they had an average of 6 years of experience. The average length of interview was 30 min (range 17–56 min). Table 1 provides a more detailed overview of interviewed physicians. Four main themes were identified: views on BSAs, factors influencing BSA prescribing and antimicrobial stewardship: practices and barriers and recommendations to improve appropriate BSA prescribing. Table 2 illustrates the identified themes and major subthemes.

### 2.1. Views on BSAs

BSAs were described by some physicians as “big guns” that can target unknown and non-specific organisms.

*“A broad-spectrum agent, I would consider those what we call them big guns of antibiotics that can actually target non-specific organism.”*

[P8, Internal medicine, 3 years experience]

Inappropriate prescribing of BSAs was defined by physicians as undercovering a suspected infection. An example was prescribing antimicrobial therapy that has no coverage for methicillin-resistant *Staphylococcus aureus* (MRSA) or *Pseudomonas* spp. for patient with hospital acquired pneumonia.

*“If someone comes with the hospital acquired pneumonia and you’re not covering for Pseudomonas or MRSA you are not covering it properly.”*

[P12, Emergency medicine, 9 years experience]

Some physicians raised concern regarding the potential impact associated with the inappropriate use of BSAs on the development of AMR.

*“A lot of patients may develop resistance and we’re not doing them any good because later on we’re going to go broader and broader till we have a resistant organism for every antibiotic and then we are stuck with nothing.”*

[P11, Orthopaedics, 1 year experience]

In addition to resistance, a concern was reported regarding the effect of BSAs on the normal microbial flora.

*“The effect of the broad-spectrum antibiotics on other healthy normal flora.”*

[P5, Neurosurgery, 1 year experience]

Furthermore, some physicians expressed concern about an individual patient’s risk for developing superinfections infections such as *Clostridioides difficile.*

*“When you use a broad-spectrum that’s usually IV or still can be oral you will subject the patient to other infections by doing that, for example, C. diff.”*

[P16, Internal medicine, 10 years experience]

### 2.2. Factors Influencing BSA Prescribing

#### 2.2.1. Patient-Related Factors

Individual patient medical history seemed to play a crucial part in the judgement to prescribe BSA. Unwell patients with co-morbidities have a risk of aggressive progression or deterioration of their illness, therefore physicians prescribe BSA to “stabilise” the patient.

*“Patients with comorbidities we suspect a very aggressive organisms or very aggressive progression of the disease so, we want to start something broad-spectrum to cover it.”*

[P10, Ear, nose and throat, 1 year experience]

Patients suffering from severe infection or sepsis that make them hemodynamic unstable are often aggressively treated with BSAs unlike stable patients with mild disease where they treated by narrower spectrum antimicrobials.

*“All comes within the signs and symptoms and clinical presentation of the patient…those who are sick, who present, for example, the severe infection or septic shock, then it would be much more appropriate to prescribe them with a broad-spectrum antimicrobial.”*

[P13, Infectious disease, 10 years experience]

#### 2.2.2. Physician-Related Factors

Physicians had a general agreement that their experiences have an influence on their BSA prescribing practices. Career progression and the accumulated clinical experiences of treating infectious disease predominantly impacted upon BSA prescribing decision-making. Two contradictory opinions were expressed, with some physicians stating that as they gained more experienced, they became aware of not prescribing BSAs when there are no indications, while others suggesting the opposite.

*“I think we tend to prescribe the same regimen to a number of patients. So, like anybody who comes in let’s say pneumonia you immediately see us prescribing azithro [azithromycin] and ceftriaxone. I think it’s [experience] heavily influenced what I’ve seen in practice”*

[P2, Internal medicine, 1.5 years experience]

*“I used to give antibiotics for example to every sore throat…Now almost zero I don’t give them unless it’s very clear there is a pus and the patient is sick, I will give them so it’s changed me a lot in my practice.”*

[P9, Emergency medicine, 16 years experience]

*“It encouraged me to use more of broad-spectrum antimicrobial therapies.”*

[P10, Ear, nose and throat, 1 year experience]

Senior physicians value their personal professional decision-making and the desire to freely select what they think to be the most suitable antimicrobial agent. This may include making BSA prescribing judgments that overrule an infectious disease specialist or clinical pharmacist recommendation.

*“I make my own decision at the end of the day because I’m the consultant you know.”*

[P15, Infectious disease, 12 years experience]

Some physicians mentioned that BSAs are overused regardless of the suspected or actual infection. It was reported that BSAs being prescribed as “analgesia”. An example was provided was the common practice of prescribing piperacillin-tazobactam for a simple urinary tract infection.

*“Usually, I come and see patient received tazocin [Piperacillin-tazobactam] even for a simple UTI, why tazocin because it is the antibiotic that we usually prescribed that what they are saying”*

[P8, Internal medicine, 3 years experience]

Physicians’ reluctance to access institution policy, to read the available guidelines and the lack of interest on antimicrobial stewardship policies were considered factors that contributed to the overuse of BSAs.

*“In our Institute there is a policy but we have a reluctance from other teams to access it…They don’t want to read the guidelines…Unfortunately most of them, they don’t read the policy.”*

[P15, Infectious disease, 12 years experience]

Physicians were confident in the diagnosis and treatment of most common presentations of bacterial infection. However, they stated there were some occasions where they tend to prescribe BSAs as a result of lacking confidence in infectious disease diagnosis. These occasions include being uncertain about the diagnosis of rare infections, dealing with infectious diseases that have similar presentations and facing difficulty distinguishing between bacterial or viral infections. An example provided was the current pandemic of Coronavirus disease of 2019 where physicians may face difficulty to differentiate between a viral or bacterial respiratory infection cause leading them to prescribe BSAs.

*“I don’t feel comfortable with the rare kind of infections especially with what’s happening now, having too many patients with pneumonia and you’re not sure if it’s actually bacteria pneumonia or viral pneumonia or atypical pneumonia so it’s yeah, sometimes it feels like I just want to make sure that time I’m not making wrong decisions.”*

[P12, Emergency medicine, 9 years experience]

Perceived risks of undertreatment by not prescribing BSAs seemed to impact upon prescribing decision. Physicians used terms such as “face our fear”, “safe”, “comfortable” and “benefit” to justify their decisions to prescribe BSAs.

*“I think people tend to feel more comfortable the more the broad-spectrum the antibiotic.”*

[P2, Internal medicine, 1.5 years experience]

#### 2.2.3. External Factors

Junior physicians recognised that their prescribing decisions were strongly impacted by senior colleagues. Some physicians agreed with their senior’s decisions on BSA prescribing and acknowledged their role in explaining and justifying the reason behind these decisions, while others described a pressure from the seniors to prescribe BSAs. Therefore, there was a trend to prescribe BSAs even if they disagreed with need for the agent.

*“Usually, my senior tries to explain why we are using this broad-spectrum rather than the other one, what’s the indication, what are the risk factors, something like that.”*

[P7, Internal medicine, 1 year experience]

*“If the consultant said give broad-spectrum and we don’t think it is correct we will follow the consultant, of course, order.”*

[P8, Internal medicine, 3 years experience]

Some physicians reported the involvement of the infectious disease (ID) specialist in every prescription of BSAs. ID specialists recognised the importance of their role on the control of BSAs prescribing and in reinforcing physicians’ knowledge.

*“ID team usually they control it more, sometimes they suggest to use it [broad-spectrum antimicrobial] sometimes they suggest to lower it down to narrow spectrum. So, I think they are very involved in the situation of using a broad-spectrum antibiotic.”*

[P10, Ear, nose and throat, 1 year experience]

Others reported that they tend to contact ID specialists only for advice on cases where they were uncertain. In contrast, positive pathogen identification from a culture, tended not to lead to interaction with an ID specialist.

*“If it’s [infection] something more oriented, proven by cultures and does not need any ID consultation I would go with the antibiotics I prescribed.”*

[P3, Orthopaedics, 2 years experience]

The cost was reported as a factor that might influence BSA prescribing. Some physicians reported considering the cost in situations where they were oriented about it.

*“Sometimes if we are oriented about it like for example, imipenem, meropenem, they are always orienting us about the price, antifungals they are always orienting us about micafungin and caspofungin and the difference in the price if we are oriented about the price and we know yes, we consider it.”*

[P14, Infectious disease, 5 years experience]

On the other hand, since working in a governmental hospital, physicians reported that they did not consider the cost while prescribing BSAs. Working in a private hospital, some physicians may consider the cost of the broad-spectrum therapy.

*“We never consider the cost in our institution.”*

[P1, Surgery, 8 years experience]

*“I only think about the cost when I work in one of the private hospitals here in Saudi, I think I try to accommodate the patient with the cheapest antibiotic that would cover properly his or her infection.”*

[P12: Emergency medicine, 9 years experience]

### 2.3. Antimicrobial Stewardship: Practices and Barriers

For suspected infections, blood and urine cultures were requested before initiating the BSA therapy. However, there were some situations where cultures were not taken. For severely ill, hypotensive and hemodynamically unstable patients, or if there was any difficulty in taking the cultures for example in getting an administration line into the patient, some physicians reported that they will not wait until they take the culture and will start the patients on a BSA immediately.

*“I think the patient is very sick, hypotensive, hemodynamically unstable. We will start even without taking the cultures. It will take time and the patient is unstable. So, we will start him on antibiotic”*

[P10, Ear, nose and throat, 1 year experience]

Moreover, it was mentioned that culture will not be requested for patient who already received the BSA therapy from other teams or departments.

*“Sometimes we receive a patient who already receive the antibiotic from emergency, from medical team, from ICU.”*

[P1, Surgery, 8 years experience]

Furthermore, for common presentation of infections where the likely causative organisms can be predicted, some physicians may not take culture for cost effectiveness.

*“Common infections with an expected organism in such cases I usually don’t send a culture I don’t send for the cost effectiveness.”*

[P5, Neurosurgery, 1 year experience]

Physicians acknowledge the practice of deescalating the BSA therapy to narrow spectrum therapy. They reported mixed views on de-escalation practices at their institution.

*“It is somehow [de-escalation] not common I would say.”*

[P7, Internal medicine, 1 year experience]

*“I would say I deescalate probably like 75% of the time.”*

[P15, Infectious disease, 12 years experience]

However, they felt uncertain about de-escalating BSA therapy to a narrow-spectrum antimicrobial when a patient is severely ill. Even if the culture indicates sensitivity to a narrow-spectrum antimicrobial, they will wait until the patient’s condition stabilises. Moreover, some patients experience fever spikes after de-escalating the therapy leading to the re-initiation of the BSA therapy.

*“Sometimes I don’t de-escalate, for example to ceftazidim because he’s still septic shock. Until the patient situations stabilised, I will consider de-escalation to the narrowest targeted antibiotic option.”*

[P15, Infectious disease, 12 years experience]

*“We have cases that we downgrade and the patient spikes the fever so, we put them again on the broader spectrum.”*

[P8, Internal medicine, 3 years experience]

Furthermore, the delay in receiving culture and sensitivity results, for example, from cultures that were taken during the weekend, is a factor for not de-escalating the BSA therapy.

*“There are scenarios when the results take a while to come out let’s say during the weekend And if you have a drug that is not sensitive to the usual antimicrobials, they do have to run more tests check for the sensitivity so that, that takes extra time There’s just one day or like two days left for that antimicrobial so sometimes, honestly, I don’t change this, which I kind of know it is wrong.”*

[P2, Internal medicine, 1.5 years experience]

It was reported that intravenous (IV) to oral switch was not common practice for hospitalised patients and patient discharged was the primary reason for considering IV to oral switch.

*“85% to 90 we are continuing the full dose IV antibiotic.”*

[P4, Orthopaedics, 5 years experience]

*“When we want the patient to go from the hospital, this is the only the only reason that we change IV to oral.”*

[P6, Orthopaedics, 4 years experience]

Several reasons of not converting IV to oral therapy where identified. Some physicians expressed the convention to maintain patients on IV antimicrobial rather than switching to oral, even if an oral form is clinically indicated.

*“I never switched from IV to oral not necessarily because it’s wrong practice, “It’s just that it’s not something that I’ve done to oral usually on discharge. From IV to oral, there’s nothing against the de-escalation from IV to oral it’s just something that we haven’t done It’s just that routinely we only change it when the patient’s for discharge but like I said, I probably, we should change it while the patient is already hospitalised for example, azithro [azithromycin] can be given oral or IV we tend to give it IV with pneumonia. There’s nothing against giving it or oral it’s just something we do.”*

[P2, Internal medicine, 1.5 years experience]

Another reason that was identified was that treating patients with IV antimicrobials gives some medics a feeling of security particularly for severely ill patients.

*“We tried just to avoid the oral although we can switch to oral but sometimes the patient sick so will not risk it and give oral while we can give IV.”*

[P14, Infectious disease, 5 years experience]

In addition, some physicians expressed a belief and evidence that IV antimicrobials held additional efficacy over oral antimicrobials.

*“We think, we believe in and we studied that that the efficacy of the IV antibiotic is much more than oral antibiotic.”*

[P1, Surgery, 8 years experience]

### 2.4. Recommendations to Improve Appropriate BSA Prescribing

Physicians identified several areas that were considered to have potential to improve the appropriate prescribing of BSAs. Physicians felt that it would be very useful to have educational sessions about BSAs such as lectures, seminars and workshops. These sessions may help them in the practice of appropriate prescribing of BSAs.

*“We must have more educational sessions about the use of antibiotics, about dealing with a sick patient, when should we use a broad-spectrum, about the disadvantage of malpractice of the usage of broad-spectrum antibiotics.”*

[P10, Ear, nose and throat, 1 year experience]

Physicians highlighted the need for a collaboration between healthcare professionals in the decision of prescribing BSAs. They suggested that there should be an involvement of multidisciplinary healthcare professionals on BSA prescribing decisions and not only having input from an ID specialist.

*“Collaboration, there should be a team for antibiotic. The privilege should be split between teams, not based on one team only ID people, antibiotic should be prescribed by all the physician who knows what to prescribed and approved by clinical pharmacists.”*

[P1, Surgery, 8 years experience]

Further, they expressed a need for the initiation of a multidisciplinary team committee to provide feedback to them about their prescribing, which can play an important role in minimising unnecessary antimicrobial prescribing.

*“We need a very active committee made up by the pharmacy, the clinical pharmacist and the infectious disease department a small committee that goes into the hot areas like emergency department, ICU, internal medicine wards and surgical wards and if they just room around they follow, we need them to follow the antibiotic prescription from different department and questioned why you have prescribed that it doesn’t work in this condition this antibiotic is usually work better”*

[P9, Emergency medicine, 16 years experience]

Guidelines were reported and considered as the ministry of practice. Physicians highlight the need to implement clinical based local guidelines that are distributed very well, to help and guide them on BSAs prescribing.

*“If there is any guideline, I would always go back to it. It says it is a mainstay of practice If there is institutional guideline that is backed up by international researchers or update, I think it will be great.”*

[P11, Orthopaedics, 1 year experience]

### 2.5. Discussion

This study explored physicians’ views, perceptions and experiences regarding the prescribing practice of BSA in a hospital setting to help optimal prescribing of BSA. It was noticed that physicians’ sense of inappropriate antimicrobial prescribing was determined more by missing or not covering a suspected infection than by avoiding negative consequences associated with over coverage such as *Clostridioides difficile* or promoting AMR. There may be many explanations of why such negative consequences were underestimated. These negative consequences may have a delayed manifestation, with a disconnect between an individual physician’s prescribing decision and its downstream impact. This affords physician anonymity and a lack of personal accountability. Moreover, being unaware or not considering such negative consequences may be reinforced by physicians not following patients up after discharge and therefore being unaware of possible readmission due to *Clostridioides difficile* or an AMR infection [17]. Furthermore, the extent to which the physicians are oriented about AMR and the impact of inappropriate prescribing on wider society may also be considered as a reason that led to the underestimation of the negative consequences of inappropriate antimicrobial prescribing. Generally, research has identified that physicians realise and perceive AMR as more of a population health or theoretical problem, and thus do not directly relate it to individual patient care [17,18].

Patients’ clinical presentations and co-morbidities were considered as a major factor in the decision to prescribe BSAs. Physicians stated that severely sick patients or patients with co-morbidities are often treated more aggressively with BSAs compared to stable patients with milder infectious disease where they are treated by narrow-spectrum antimicrobials. A qualitative study conducted with 40 general medical practitioners to explore factors that influence their decisions to prescribe BSAs, particularly fluoroquinolones, rather than a narrower spectrum antimicrobial [19]. Of the factors that were reported to influence the decision to prescribe BSAs, patient presenting condition was considered a major factor to prescribe BSAs, where general medical practitioners justified such prescribing practices to prevent significant clinical decline [19].

Physicians had a general agreement that their experiences have an influence on their BSA prescribing. With career progression through the medical profession, individual experiences had considerable impact on BSA prescribing. This was in agreement with other studies [20,21].

Diagnostic uncertainty was reported to drive BSA prescribing. This is similar to what has been previously reported in the literature [22,23,24]. In the case of diagnostic uncertainty in infectious disease, physicians seek the reassurance of prescribing antimicrobials, particularly BSAs. While such a decision is understandable and indeed reasonable for a patient with suspected sepsis, physicians also have a tendency to prescribe BSAs in stable patients where narrow-spectrum antimicrobials would be more clinically justified. The impact of uncertainty avoidance on antimicrobial prescribing has been identified in other qualitative studies conducted in hospital settings [17,25] as an approach to prevent the patient deterioration [26]. This may explain the variability in antimicrobial prescribing that is identified between different countries [27,28].

Physicians felt that prescribing BSAs removed their fear and insecurity. Livorsi et al. [17] conducted a qualitative study to identify factors that influence physicians’ antimicrobial prescribing decisions in an inpatient setting. They found that physicians tend to prescribe BSAs to assuage their fear of missing any unidentified organism(s) [17]. In this study, the perceived risk of not prescribing BSAs overruled longer-term public health risks. Prior studies also described similar immediate pressures in antimicrobial decision-making [29,30]. Such dynamics are considered to be significant in antimicrobial prescribing in hospital settings, strongly favouring antimicrobial over-prescribing over the longer-term public health considerations.

Junior physicians reported that senior physicians strongly impacted upon their BSA-prescribing decisions. They described pressure from the seniors to prescribe BSAs even if they disagreed with need for such a prescription. Qualitative studies from the UK, USA, Belgium and Ireland have also recognised senior physician pressure as a significant contributing factor on antimicrobial prescribing, which overrules the impact of local and national guidelines and policies [17,20,31,32,33]. According to current findings, attempts to improve antimicrobial prescribing in inpatient settings have to acknowledge the influence of decision-making hierarchy.

Several studies have identified that antimicrobial cost influences antimicrobial prescribing [34,35]. In the current study, some, but not all, physicians identified antimicrobial cost as a factor that might influence BSA prescribing. Governmental hospital physicians less frequently consider antimicrobial cost as care is financed by the state [36].

De-escalation is promoted to delay the development of bacterial resistance [37]. This approach involves changing the empiric BSAs to a narrower spectrum following positive microbiology identification and is associated with no clinical detriment [37]. Qualitative examination of barriers to antimicrobial therapy de-escalation found that physicians still felt uncertainty about de-escalating BSAs to a narrow-spectrum antimicrobial after receiving culture results, especially for severely ill patients. This was exemplified by their attitude of “never change a winning team”. Moreover, organisational constraints where a delay in obtaining the culture results also lessens the likelihood of de-escalation. In a patient responding to initial therapy, de-escalation is postponed until senior clinical review, prolonging patient duration on BSAs [38]. The present study found similar findings. Additionally, the periodic unavailability of the narrow antimicrobials sometimes exacerbated de-escalation.

Occasionally, a clinical driver for the intravenous to oral switch was to facilitate clinical discharge of the patient from hospital. However, there was a preference to not undertake IV to oral switching. Reasons identified included the convenience/habit of maintaining patients on IV antimicrobials; a sense of security provided from maintaining the intravenous route, particularly in clinically fragile patients; and the belief that IV forms of antimicrobials are more effective than oral forms. The latter, erroneous belief has been previously reported [39].

A number of recommendations were made by the interviewed physicians to improve the appropriate practice of BSAs prescribing in Saudi Arabia. These include additional education, prescribing feedback, multidisciplinary team decision-making and local guideline implementation. These recommendations are in line with the Saudi national action plan for minimising AMR [40].

Our study adds to the existing evidence regarding why and how physicians decided to prescribed antimicrobials particularly BSAs by incorporating the perspectives of a wide range of physicians in terms of specialties and years of experience. Moreover, in the Middle East, antimicrobials are reportedly overused [41], stewardship is less developed [42] and this is providing baseline data for such a system to be better developed. Stewardship is a global issue, and we are all only as secure as the weakest point in the link.

To the knowledge of the researchers, this is the first qualitative study to explore physicians’ views and perceptions about BSAs and identify factors influencing BSAs prescribing practices in KSA. The study was validated in terms of the quality of the collected data and the data analysis where it was reviewed by an external researcher who is expert in the field. Trustworthiness of the qualitative study was also ensured by using the COREQ guideline [43].

The study had some limitations. It was conducted in a single tertiary care hospital and therefore may not be representative of physicians’ views and general BSA prescribing practices. While physicians have various levels of seniority and thematic saturation was identified, there is a possibility that minor views and perceptions may have been missed due to being unable to interview physicians from a broader range of specialties. A broader exploration of physicians’ views and perceptions in different hospitals including private hospitals would be valuable and is anticipated in future studies. Moreover, physicians interested in the topic may be more likely to participate in the study. This is always an inherent risk of purposive sampling or voluntary participation. However, the views are broadly aligned with that reported from other countries. Finally, given the bias associated with self-reporting, although this bias has been minimised by the use of telephone interviews instead of face-to-face interviews, there is a risk of social desirability bias where physicians provide a more socially acceptable response [44,45]. During the interview, it became evident that physicians felt comfortable and that their prescribing practices were not being assessed or judged. However, their statements reflecting that BSAs are prescribed inappropriately is evidence of that.

## 3. Materials and Methods

### 3.1. Study Design and Setting

Qualitative semistructured telephone interviews were conducted from April to June 2020 with physicians in a single tertiary care hospital in Riyadh, Saudi Arabia. The reason for conducting telephone interviews was the emergence of the coronavirus pandemic crisis. Evidence recommends that telephone interviews may provide data with similar quality to face-to-face interviews [46,47]. Semistructured interviews represent a commonly used method for qualitative data collection in healthcare research [48]. This type of interview has numerous advantages, including its ability to provide an exploration and investigation participants’ and researchers’ agendas [49]. Furthermore, it allows for a deep understanding of the participants’ knowledge, views or experiences in a field of interest [50]. The study was reported following the Consolidated Criteria for Reporting Qualitative Studies (COREQ) reporting guideline [43] (Supplementary S1).

### 3.2. Interview Topic Guide

The interview topic guide was developed following a thorough review of the literature. In addition to the consideration of the main findings of previously conducted drug utilisation study at the same hospital [51]. The validation of the interview topic guide was obtained before the start of the study. It was reviewed and validated by all co-authors (A.B.M., H.A. and A.M.). Furthermore, it was reviewed by an expert qualitative researcher with relevant background and by one infectious disease clinical pharmacist specialist. The interview topic guide was tested in pilot interviews with two healthcare professionals and only minor amendments were made according to their feedback. The interview topic guide was comprised of two main parts: The first part was about physicians’ demographics, while the second part was the main questions which was further divided into three sections; physicians’ practices of BSAs prescribing, challenges and barriers of appropriate BSAs prescribing and recommendations to improve BSAs prescribing practice (Appendix A).

### 3.3. Participant Enrolment

The study participants involved physicians who prescribe BSAs for adult hospitalised patients. Purposeful sampling was employed as the sampling strategy where the physicians were approached based on their experience in BSAs prescribing for adult hospitalised patients as any physician who prescribed BSA was invited to participate on the study (Supplementary S2). A snowball sampling strategy was used as interviewed physicians were asked if they have colleagues or peers who prescribe BSAs and interested to take part in the study. Participations in the study was voluntary and all physicians reviewed and signed an electronic consent form after reviewing the participants information sheet prior to the start of the interview (Supplementary S3 and S4). Sample size was not decided until data saturation was achieved, where no new views or ideas emerged [52,53].

### 3.4. Qualitative Analysis

All interviews were audio recorded and transcribed verbatim. Transcribed files were checked and screened for accuracy. Data were organized and stored using a computer software to manage qualitative data (NVivo)^®^ v12, QSR International. 2018. A thematic data analysis approach was used, following the six-step model suggested by Braun and Clarks [54]. To check the reliability of the coding, data were independently checked and verified by a qualitative research expert, who reviewed a random sample of 20% of the transcripts and agreement on identified codes and themes was achieved.

## 4. Conclusions

This study emphasises the need for a multifaceted intervention to improve the appropriateness of BSAs prescribing. Attempts to encourage physicians’ prudent and appropriate prescribing of BSAs must take into account factors related to patients, physicians and the institution.

## Figures and Tables

**Table 1:** Characteristic of interviewed physicians.

Physician Number	Age (Years)	Specialty	Working Position (Years)	Working Experience
1	32	Surgery	Fellow	8
2	26	Internal medicine	Junior resident	1.5
3	25	Orthopaedics	Junior resident	2
4	27	Orthopaedics	Senior resident	5
5	25	Neurosurgery	Junior resident	1
6	27	Orthopaedics	Senior resident	4
7	26	Internal medicine	Junior resident	1
8	28	Internal medicine	Senior resident	3
9	40	Emergency medicine	Consultant	16
10	26	Ear, nose and throat	Junior resident	1
11	27	Orthopaedics	Junior resident	1
12	37	Emergency medicine	Consultant	9
13	43	Infectious disease	Consultant	10
14	30	Infectious disease	Fellow	5
15	36	Infectious disease	Consultant	12
16	35	Internal medicine	Consultant	10

**Table 2:** Identified themes and subthemes.

Theme	Subtheme
Views on BSAs	Physicians’ perceptions of BSAs Concern for AMR and other drawbacks associated with BSAs
Factors influencing BSA prescribing	Patient-related factors: Medical history Clinical presentation and the severity of the infection
	Physician-related factors: Physician experience Habit and decision-making autonomy Over prescribing behaviours Anxiety and fear
	External factors: The influence of the medical hierarchy Role of the infectious disease specialist Cost of antimicrobial agents
Antimicrobial stewardship: practices and barriers	Taking culture before administering the BSA therapy De-escalation therapy Intravenous to oral switch
Recommendations to improve appropriate BSA prescribing	Education, awareness and training Audit and feedback Guideline implementation Multidisciplinary decision making

## Supplementary Materials

The following are available online at https://www.mdpi.com/article/10.3390/antibiotics10040366/s1, Document S1: The COREQ checklist; Document S2: Participants’ invitation letter; Document S3: Participants’ information sheet; Document S4: Consent form.

## Appendix A

### Appendix A.1. Interview Topic Guide

#### Appendix A.1.1. Practice of Broad-spectrum Antimicrobial Prescribing

1. What would you consider a broad-spectrum agent?
2. What is your understanding of appropriate broad-spectrum antimicrobial prescribing?
3. What is your understanding of inappropriate broad-spectrum antimicrobial prescribing?
4. In your daily practice, how do you plan or decide on prescribing broad-spectrum antimicrobials?
5. In your daily practice, how often do you request cultures before starting broad-spectrum antimicrobial therapy? In which situation(s) do you decided not to request a culture prior to initiation of a broad-spectrum antimicrobial?
6. What factors influence your decision to start broad- spectrum antimicrobial?
7. In your practice, have you ever prescribed broad-spectrum antimicrobial when you think you could prescribe a narrow spectrum? Tell me about those circumstances?
8. In your daily practice, how often do you subsequently narrow antimicrobial therapy based on culture/sensitivity results?
9. In your daily practice, how often do you convert antimicrobial therapy from IV to oral?
10. How has your clinical experiences shape your practice of prescribing broad-spectrum antimicrobials?
11. How has the clinical experience of your colleagues’ practice has impacted on your broad-spectrum antimicrobial prescribing?
12. How has the institutional policy impacted your broad-spectrum antimicrobial prescribing practice?
13. How has the institutional support i.e., infectious diseases specialist, clinical pharmacist and education impacted your broad-spectrum antimicrobial prescribing practice?

#### Appendix A.1.2. Barriers of Appropriate Broad-spectrum Antimicrobial Prescribing

1. How do you view your broad-spectrum antimicrobial prescribing practices compared to your colleagues? Do you agree with their decision on broad-spectrum antimicrobial prescribing? how are disagreements on therapy discussed/concluded?
2. In your own view and clinical experience, what could be the possible challenges/barriers associated with broad-spectrum antimicrobial prescribing?
3. In your belief, who or what contribute to these challenges/barriers?

#### Appendix A.1.3. Strategies and Interventions to Improve Broad-spectrum Antimicrobial Prescribing

1. In your daily practice, do you use any antimicrobial guidelines to help you in your antimicrobial prescribing, if yes what is/are they?
2. In your daily practice, do you use any electronic tools to help you in your antimicrobial prescribing, if yes what is/are they?
3. Do you think you have had sufficient support, education and training on broad-spectrum antimicrobial prescribing?
4. In your view, what could be the most useful tool(s), intervention(s) or measure(s) to improve appropriate broad-spectrum antimicrobials prescribing?

### Appendix A.2. Summary

- Is there anything else you would like to add?

### Appendix A.3. Probing and Prompting

- Can you tell me more about?
- What do you mean by that?
- Can you please give me an example?
- Could you explain more?
- Is there anything you want to say about this?
