# Supplementary material for: Exploring Physicians’ Views, Perceptions and Experiences about Broad-Spectrum Antimicrobial Prescribing in a Tertiary Care Hospital Riyadh, Saudi Arabia: A Qualitative Approach
Source: Antibiotics (Basel). 2021 Mar 31;10(4):366. doi: 10.3390/antibiotics10040366 (PMC8067237; doi:10.3390/antibiotics10040366)
Supplement: Supplementary file 1 [file antibiotics-10-00366-s001.zip › Supplementary/Supplementary 3 Participant information sheet.docx]

# Supplementary 3: Participant’s information sheet

**Title of the study**

Exploring Physicians’ Views, Perceptions and Experiences about Broad-Spectrum Antimicrobial Prescribing in a Tertiary Care Hospital Riyadh, Saudi Arabia: A Qualitative Approach

# Purpose of the study

The aim of this study is to understand broad-spectrum antimicrobial prescribing practises among physician practicing in hospital settings, in order to find measures that could improve broad-spectrum antimicrobial prescribing practices.

# Why am I being asked to participate in this research?

We are recruiting physicans who have prescribed broad-spectrum antimicrobails for adult hospitalised patients.

# What will happen if I decide to participate?

If you decide to participate, a PhD student, Nada Alsaleh (NA), will conduct a short interview which will last approximately 30 minutes to explore your views, perceptions and practice in broad-spectrum antimicrobial prescribing. The interview will be conducted at your convenient date and time. You can stop the interview any time if you no longer want to participate or you can skip any question that you don’t want to answer. The interview will be audio recorded. Before starting the interview, you will be given a consent form to sign. You can have a copy of the signed consent form and this information sheet.

# Do I have to take a part?

No, your participation is voluntary and you are free to decide not to participate or to withdraw at any time without giving a reason.

# Is there is any possible risk of participating?

All data will be confidential and will remain anonymous.

# What are the possible benefits of participating?

Participating will provide information that will help to get better understanding of the broad- spectrum antimicrobial prescribing practices. In turn, this will help in developing a quality improvement plan that will improve patients’ outcome and decrease antimicrobial resistance. The findings will be reported in the researcher’s (NA) PhD thesis and may be published in journals.

**Contact for further information:** If you need more information, you can contact the researcher, Nada Alsaleh (who will conduct the interview) using the following contact details:

Nada Alsaleh

PhD Research Student

Institute of Pharmacy and Biomedical Science University of Strathclyde Email[:Nada.alsaleh@strath.ac.uk](mailto:Nada.alsaleh@strath.ac.uk)

Tel: 966555443885

# Thank you for reading this information sheet and for considering participating in this research
