# Supplementary material for: Exploring Physicians’ Views, Perceptions and Experiences about Broad-Spectrum Antimicrobial Prescribing in a Tertiary Care Hospital Riyadh, Saudi Arabia: A Qualitative Approach
Source: Antibiotics (Basel). 2021 Mar 31;10(4):366. doi: 10.3390/antibiotics10040366 (PMC8067237; doi:10.3390/antibiotics10040366)
Supplement: Supplementary file 1 [file antibiotics-10-00366-s001.zip › Supplementary/Supplementary 2 Participant’s invitation letter.docx]

## Supplementary 2: Participant’s invitation letter

You are invited to participate in this study which is investigating the physicians' practices in broad-spectrum antimicrobials prescribing in your hospital. It is concerned with determining physicians’ practices of broad-spectrum antimicrobials and exploring what influences them when prescribing broad-spectrum antimicrobials. This study will help to inform future developments associated with the delivery of care in your hospital. The aim is to find measures that could help physicians’ when prescribing broad-spectrum antimicrobials.

All physicians’ prescribing broad-spectrum antimicrobial to adult patients and working in hospital wards are welcomed to take part in this research.

This is qualitative research; the interview will take approximately 30 minutes and will be digitally recorded.

If you are willing to participare, please email me and I will be happy to schedule a meeting with you at your convenience date and time. Kindly read the attached participant’s information sheet, and if you require any further information or clarification, please contact me

Thank you for your time and consideration.

Yours faithfully,

Nada Alsaleh,

Institute of Pharmacy and biomedical Science

University of Strathclyde

Email:[Nada.alsaleh@strath.ac.uk](mailto:Nada.alsaleh@strath.ac.uk)
