# Supplementary material for: Exploring Physicians’ Views, Perceptions and Experiences about Broad-Spectrum Antimicrobial Prescribing in a Tertiary Care Hospital Riyadh, Saudi Arabia: A Qualitative Approach
Source: Antibiotics (Basel). 2021 Mar 31;10(4):366. doi: 10.3390/antibiotics10040366 (PMC8067237; doi:10.3390/antibiotics10040366)
Supplement: Supplementary file 1 [file antibiotics-10-00366-s001.zip › Supplementary/Supplementary 1COREQ.docx]

Supplementary 1: COREQ: Consolidated criteria for reporting qualitative research: a 32-item checklist for interviews and focus groups

Developed from:

Tong A, Sainsbury P, Craig J. Consolidated criteria for reporting qualitative research (COREQ): a 32-item checklist for interviews and focus groups. *International Journal for Quality in Health Care*. 2007. Volume 19, Number 6: pp. 349 – 357

| **Section/Topic** | **Item**  **No** | **Checklist item** | **Reported on**  **page No** |
| --- | --- | --- | --- |
| **Domain 1: Research team and reﬂexivity** | | | |
| Personal Characteristics | | | |
| Interviewer/facilitator | 1 | Which author/s conducted the interview or focus group?  Interviewer/facilitator | Nada Alsaleh |
| Credentials | 2 | What were the researcher’s  credentials? E.g. PhD, MD | MSc pharmacist |
| Occupation | 3 | What was their occupation at the time  of the study? | PhD student |
| Gender | 4 | Was the researcher male or female? | Female |
| Experience and training | 5 | What experience or training did the researcher have? | The researcher participated in many qualitative research courses  and workshops |
| Relationship with participants | | | |
| Relationship established | 6 | Was a relationship established prior to  study commencement? | No |
| Participant knowledge of the interviewer | 7 | What did the participants know about  the researcher? e.g. personal goals, reasons for doing the research | Participant  information sheet |
| Interviewer characteristics | 8 | What characteristics were reported about the interviewer/facilitator? e.g. Bias, assumptions, reasons and  interests in the research topic | Not reported |
| Domain 2: study design | | | |
| Theoretical framework | | | |
| Methodological orientation and Theory | 9 | What methodological orientation was stated to underpin the study? e.g. grounded theory, discourse analysis, ethnography, phenomenology, content analysis | Methods |
| Participant selection | | | |
| Sampling | 10 | How were participants selected? e.g.  purposive, convenience, consecutive, snowball | Purposive and snowball detail |

|  |  |  | description in  the methods |
| --- | --- | --- | --- |
| Method of approach | 11 | How were participants approached?  e.g. face-to-face, telephone, mail, email | Email |
| Sample size | 12 | How many participants were in the  study? | 16 |
| Non-participation | 13 | How many people refused to  participate or dropped out? Reasons? | 0 |
| Setting of data collection | 14 | Where was the data collected? e.g. home, clinic, workplace | Data was collected via  phone |
| Presence of non-  participants | 15 | Was anyone else present besides the  participants and researchers? | No |
| Description of sample | 16 | What are the important characteristics of the sample? e.g. demographic data, date | Results Table 1 speciality and year of  experience |
| Data collection | | | |
| Interview guide | 17 | Were questions, prompts, guides provided by the authors? Was it pilot  tested? | Yes |
| Repeat interviews | 18 | Were repeat interviews carried out? If  yes, how many? | No |
| Audio/visual recording | 19 | Did the research use audio or visual  recording to collect the data? | audio recorded |
| Field notes | 20 | Were ﬁeld notes made during and/or  after the interview or focus group? | No |
| Duration | 21 | What was the duration of the interviews or focus group? | An average duration of 30 minutes [durations ranged from 17-  56 minutes] |
| Data saturation | 22 | Was data saturation discussed? | Yes |
| Transcripts returned | 23 | Were transcripts returned to participants for comment and/or  correction? | No |
| Domain 3: analysis and findings | | | |
| Data analysis | | | |
| Number of data coders | 24 | How many data coders coded the data? | Methods |
| Description of the coding  tree | 25 | Did authors provide a description of  the coding tree? | No |
| Derivation of themes | 26 | Were themes identiﬁed in advance or  derived from the data? | Derived from the  data |
| Software | 27 | What software, if applicable, was used  to manage the data? | Nvivo |

| Participant checking | 28 | Did participants provide feedback on  the ﬁndings? | No |
| --- | --- | --- | --- |
| Reporting | | | |
| Quotations presented | 29 | Were participant quotations presented to illustrate the themes / ﬁndings? Was each quotation identiﬁed? e.g.  participant number | Yes-results |
| Data and ﬁndings  consistent | 30 | Was there consistency between the  data presented and the ﬁndings? | Yes-discussion |
| Clarity of major themes | 31 | Were major themes clearly presented  in the ﬁndings? | Yes-results |
| Clarity of minor themes | 32 | Is there a description of diverse cases  or discussion of minor themes? | No |
