# Supplementary material for: Exploring Physicians’ Views, Perceptions and Experiences about Broad-Spectrum Antimicrobial Prescribing in a Tertiary Care Hospital Riyadh, Saudi Arabia: A Qualitative Approach
Source: Antibiotics (Basel). 2021 Mar 31;10(4):366. doi: 10.3390/antibiotics10040366 (PMC8067237; doi:10.3390/antibiotics10040366)
Supplement: Supplementary file 1 [file antibiotics-10-00366-s001.zip › Supplementary/Supplementary 4 Consent form.docx]

Supplementary 4: Consent form

Study title: Exploring Physicians’ Views, Perceptions and Experiences about Broad-Spectrum Antimicrobial Prescribing in a Tertiary Care Hospital Riyadh, Saudi Arabia: A Qualitative Approach

| I confirm that I have read the participant information sheet and fully understand the  information provided |
| --- |
| I confirm that I was given the opportunity to ask questions |
| I understand that my participation in this study is voluntary and that I am free to  withdraw at any time without giving reasons |
| I understand that the interview will be audio recorded then transcribed |
| I understand that the data obtained from the interview will be anonymised |
| I understand that the results may be published |
| I agree to take part in the study and participate in the interview |

|  | **_ _ / _ _ / _ _ _ _** |  |
| --- | --- | --- |
| **Name of participant** | **Date** | **Signature** |
|  | **_ _ / _ _ / _ _ _ _** |  |
| **Name of researcher** | **Date** | **Signature** |
